# The ethnic distribution of sickle cell disease in Sudan

**DOI:** 10.11604/pamj.2014.18.13.3280

**Published:** 2014-05-03

**Authors:** Majdi Mohammed Sabahelzain, Hanan Hamamy

**Affiliations:** 1 Reproductive and Child Health Research Unit, University of Medical Sciences and Technology, Khartoum, Sudan; 2 Department of Genetic Medicine and Development Geneva University, Geneva, Switzerland

**Keywords:** Sickle cell anemia, sickle cell gene, haplotype, Sudan, Messeryia, tribe, Haemoglobinopathy, Kordofan, Darfur, Khartoum

## Abstract

Sickle cell disease (SCD) is one of the most common inherited disorders of haemoglobin in Africa and it is expected that sickle cell trait varies in frequency in different areas in Sudan. An extensive literature search was carried out accessing the US National Library of Medicine, the WHO Eastern Mediterranean Region resources, the Catalogue for Transmission Genetics in Arabs and papers and documents published in Sudan that included data on the prevalence of sickle cell anaemia and trait. Rates of SCA and trait varied in different areas in Sudan with the highest rates reported from Western and Eastern Sudan where one in every 123 children born in Messeryia tribe in Western Sudan is at risk of having SCD. High consanguinity rates and malaria endemicity are strong related factors with sickle cell gene in Sudan. This review will present what is known about the rates of sickle cell gene in different ethnic groups in Sudan.

## Introduction

**Figure 1:**
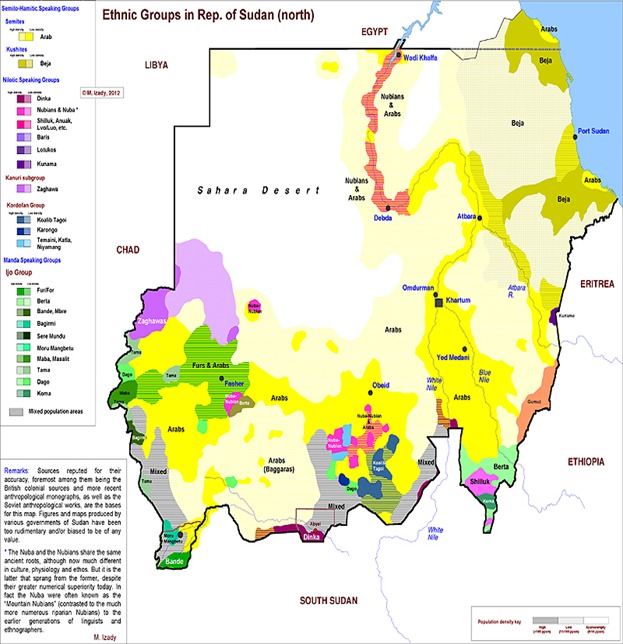
Map of Sudan showing different areas and the distribution of ethnic groups (From http://nealrauhauser.wordpress.com/2012/12/25/sudan-africas-yugoslavia/sudanethnicgroups/)

Sudan includes variable ethnic groups that range from Arabs to African and Afro-Arabs tribes. These ethnic groups include groups with Negroid genetic characteristics with an established history in the area such as Nuba and Nilotes. Other groups include Arab, Hausa and Copt who migrated to the area in different times in history, as well as the Arab-negroid admixture tribes [1, 2]. A study was carried out to analyze genetically Fur, Beja, Gaalin, Hawazma, and Messeryia tribes, which belong to different ethnic and linguistic groups in Sudan. Fur tribe has been found to have intermediate genetic characteristics between the Arabs and Negroids. Beja and Gaalin tribes have more Arab genetic characteristics when compared to Hawazma, and Messeryia tribes which have more Negroid admixture [2] (Figure 1). With the diversity in ethnic groups, Sudan has a total of 133 different local languages that belong to three major African linguistic families, the Niger-Congo, Nilo-Saharan and Afro-Asiatic language families. [3] The ethnic diversity, rapid increase in the population, high fertility rate, large family size, high consanguinity rate and the historical, cultural, traditional and religious background of these ethnic groups, highlight the interest of genetic studies in Sudan. First cousin marriage rates in Sudan are amongst the highest worldwide reaching 40-45% of all marriages [4–6]. This review presents the rates of sickle cell gene in different ethnic groups in Sudan. The haemoglobinopathies are inherited as autosomal recessive (AR) disorders, where carrier parents could transmit the abnormal genes to their offspring. If both, the father and mother are heterozygotes for HbS, there is chance of 25% of having a homozygous HbSS (Sickle cell anemia, SCA) child. If one parent is a carrier for HbS and the other is carrier for one of the abnormal hemoglobins, it results in a double heterozygote state. Heterozygotes are generally asymptomatic carriers (traits), while the SCD is expressed in the homozygotes and the double heterozygotes for two abnormal haemoglobin genes or HbS and the thalassaemias.

## Methods

An extensive literature search was carried out accessing the US National Library of Medicine [7], and the WHO resources including the Index Medicus for Eastern Mediterranean Region [8]. Keywords used for the search included “Sudan” combined with each of the following search terms: Sickle cell anaemia, sickle cell trait, haemoglobinopathy. The Catalogue for Transmission Genetics in Arabs [9], a database on genetic disorders in Arab populations maintained by the Centre for Arab Genomic Studies (CAGS) was also searched for relevant data. Papers and documents published in Sudan that included data on the prevalence of sickle cell anaemia and trait were searched and cited. There are no exclusion criteria for citing published data because of the general dearth of studies on sickle cell anaemia in Sudan.

## Results

### Sickle cell gene in Sudan

**The Origin of Sickle cell gene in Sudan**: Based on analysis of Y-chromosome haplogroups, the sickle cell gene may have been preferentially introduced through males of migrating West African tribes, particularly Hausa-Fulani, and Bagara in the large migrations that began in the eighteenth century and escalated during the nineteenth and early twentieth century [10]. The haplotypes associated with the S gene in Sudan are most likely to be the Cameroon, Benin, Bantu and Senegal haplotypes rather than Saudi-Asian haplotype. Among 40 clinically and electrophoretically confirmed SCA cases, the Cameroon and Benin haplotypes accounted for 25% each of the samples [11]. The most frequent haplotype among 143 chromosomes with S gene was the Cameroon (35.0%), followed by the Benin (29.4%), the Senegal (18.2%) and the Bantu (2.8%). The Indian-Arab haplotype was not observed. Three atypical haplotypes were identified in 17 patients, occurring at a combined frequency of 14.6%. One of these, found at the high frequency of 11.8%, possibly represented a new Sudan haplotype [12].

### Occurrence and distribution of sickle cell anemia among the Sudanese

In 1950, the first case of HbS gene was reported in Sudan [13]. Later studies showed that sickle cell gene frequencies vary from region to another in Sudan as well as within the same region.

***Central Sudan***: Sickle cell gene is known to be prevalent in the Khartoum area, which is the capital of the country and situated in central Sudan. In the 1980s when drought and famine struck western Sudan, a huge number of migrations took place and many tribes settled around Khartoum. This unique situation made Khartoum a multiethnic area, with a blend of almost all the Sudanese tribes. Among 632 patients attending various clinics at the Khartoum Teaching Hospital, there were 5.1% with Hb AS and 0.8% with Hb SS [14]. Sickle cell disease is the major haemoglobinopathy seen in the Khartoum, the capital of Sudan. This may be attributed to the migration of tribes from western Sudan as a result of drought and desertification in the 1970s and 1980s, and the conflicts in Darfur in 2005. The rate is highest in Western Sudanese ethnic groups particularly in Messeryia tribes in Darfur and Kordofan regions [15]. In the Blue Nile area, where groups of indigenous population live, the prevalence ranges from 0-5% in addition to a rate of 16% among some immigrant tribes from western Sudan and West Africa in the area [14, 15]. The SCA presentation is usually severe and accompanied with major complications, and could be fatal in early childhood [13].

***Northern Sudan***: Although the data about sickle cells gene in the north of Sudan is incomplete, it seems that this area shows a low frequency of SCA. A study conducted in the north of Sudan in Shagia and Manaseer tribes confirmed that the sickle cell gene is lower in the north of Sudan than in other areas [16]. Shagia are partly nomadic, isolated, and an agricultural population. Therefore, it is difficult to determine significantly whether they are Arab or African. Manaseer tribe is of Arab origin. Both of them inhabit the 4th cataract region. [16]

***Eastern Sudan***: Eastern region of Sudan is composed of three states, Gedarif, Kasala, and Red Sea states. Most studies on sickle cell gene done in this region were conducted in Gedarif state, since the majority of the population migrated to and settled in this state in different decades in the past century. Blood samples tested for SCA among 100 individuals from different tribes in Gedarif state showed that 20 samples had HbSS, 55 samples had HbAS and 25 samples had HbAA. The results of this study cannot be generalized for the population of the area due to the low sample number [17]. Among the population of the same area, a higher number of individuals were studied (261 from Hausa and 285 from Massaleet tribes), showing that sickle cells trait Hb AS was found in approximately 35% of study subjects in Hausa and 24% in Massaleet, whereas Hb SS was reported as 6% and 5% in Hausa and Massaleet respectively [18].

***Western Sudan***: The presence of HbS is already well documented among Kordofan and Darfur region inhabitants, especially Albaggara, an Afro-Arab constellation of tribes with a predominantly African descent [19]. Some findings of a study conducted in Elobied hospital in north Kordofan state, showed that sickle cell trait in relatives of patients suffering from sickle cell disease (SCD) who were referred to this Hospital, was 54% of target samples, which concentrated mainly in two tribes, Bederia and Fulani. Sickle cell disease in Messeryia of Darfur and Messeryia Hummer of Kordofan showed a prevalence of 30.4% and 18% respectively. It is estimated that one in every 123 children born in Messeryia tribe is at risk of having SCD [14]. Many indigenous tribes that inhabit Darfur region and belong to the Negroid ethnic group and are a part of Nilo-Saharan language family such as the Berge, Fur and Masaleet had the highest frequencies of the S gene among them [11].

## Discussion

This review of published data on the prevalence of SC trait and disease in Sudan showed the dearth of studies addressing this issue. The available data revealed the wide range of SC disease frequencies in different areas of Sudan ranging from 0.8% in central Sudan to 30.4% in Western Sudan. The Messeryia tribe (a branch of the Baggara tribes) in Kordofan and Darfur showed the highest rate of sickle cell disease where it is estimated that one in every 123 children born is at risk of having SCD. Gedarif state in Eastern Sudan also showed high rate of sickle cell gene among the population that migrated from Western Africa and Sudan. The high prevalence of sickle cell gene in Sudan could be attributed to the following factors: Malaria is endemic in Sudan. The advantage of sickle cell trait (Hb AS) and its strong association with protection against all forms of clinical P. falciparum malaria is well established. Heterozygotes for the sickle gene (AS) are relatively protected against the danger of dying of malaria through the plausibile mechanism that in AS heterozygotes P falciparum-infected red cells sickle preferentially and are then removed by macrophages. The clinically relevant consequence of this process is to keep parasitemia relatively low in AS heterozygotes, [20]; The high consanguinity rates in Sudan and the rate of first cousin marriages is the highest when compared with the other Arab countries (which exceeds 40%), which could increase the prevalence of autosomal recessive diseases such as SCD. Moreover, the traditional tribal society is still existent in Sudan [21]; The lack of public health measures and services for the prevention of genetic disorders in general; The selective termination of pregnancy of an affected fetus is illegal in Sudan.

## Conclusion

There is a need to initiate systematic epidemiological studies to assess the prevalence rates of SCD and SCT in different areas in Sudan. Recently, many Arab countries have ongoing premarital screening programs for haemoglobinopathies, with success stories of reducing the birth rate of affected with SCD, such as in Bahrain [22]. It is recommended that such programs for premarital screening of haemoglobinopathies are initiated in Sudan, especially in known areas with high prevalence rates of SCA such as Darfur and Kordofan regions, to allow couples to take an informative decision when they are both carriers of the gene.
